# Humanin, a Cytoprotective Peptide, Is Expressed in Carotid Artherosclerotic Plaques in Humans

**DOI:** 10.1371/journal.pone.0031065

**Published:** 2012-02-06

**Authors:** David G. Zacharias, Sung Gyun Kim, Alfonso Eirin Massat, Adi R. Bachar, Yun K. Oh, Joerg Herrmann, Martin Rodriguez-Porcel, Pinchas Cohen, Lilach O. Lerman, Amir Lerman

**Affiliations:** 1 Division of Cardiovascular Diseases, Department of Nephrology and Hypertension, Mayo Clinic College of Medicine, Rochester, Minnesota, United States of America; 2 Department of Nephrology and Hypertension, Mayo Clinic College of Medicine, Rochester, Minnesota, United States of America; 3 Department of Pediatrics, Division of Endocrinology, Mattel Children's Hospital, University of California, Los Angeles, Los Angeles, California, United States of America; Uppsala University, Sweden

## Abstract

**Objective:**

The mechanism of atherosclerotic plaque progression leading to instability, rupture, and ischemic manifestation involves oxidative stress and apoptosis. Humanin (HN) is a newly emerging endogenously expressed cytoprotective peptide. Our goal was to determine the presence and localization of HN in carotid atherosclerotic plaques.

**Methods and Results:**

Plaque specimens from 34 patients undergoing carotid endarterectomy were classified according to symptomatic history. Immunostaining combined with digital microscopy revealed greater expression of HN in the unstable plaques of symptomatic compared to asymptomatic patients (29.42±2.05 vs. 14.14±2.13% of plaque area, p<0.0001). These data were further confirmed by immunoblot (density of HN/β-actin standard symptomatic vs. asymptomatic 1.32±0.14 vs. 0.79±0.11, p<0.01). TUNEL staining revealed a higher proportion of apoptotic nuclei in the plaques of symptomatic patients compared to asymptomatic (68.25±3.61 vs. 33.46±4.46% of nuclei, p<0.01). Double immunofluorescence labeling revealed co-localization of HN with macrophages (both M1 and M2 polarization), smooth muscle cells, fibroblasts, and dendritic cells as well as with inflammatory markers MMP2 and MMP9.

**Conclusions:**

The study demonstrates a higher expression of HN in unstable carotid plaques that is localized to multiple cell types within the plaque. These data support the involvement of HN in atherosclerosis, possibly as an endogenous response to the inflammatory and apoptotic processes within the atheromatous plaque.

## Introduction

Atherosclerosis is a progressive and inflammatory disease characterized by well-defined lipid plaques commonly associated with necrotic cores, calcified regions, and inflammatory cells [1]. Infiltrating smooth muscle cells, fibroblasts, and immune cells (notably macrophages but also dendritic cells, T and B cells, neutrophils, and mast cells) are involved in plaque progression and destabilization [2], [3], [4], [5].

Cellular apoptosis occurs within the atheroma and is well-documented yet its role is not fully understood. A number of proapoptotic factors are documented including reactive oxygen species (ROS) oxidant stress, oxidized low-density lipoprotein (Ox-LDL), elevated TNF-α, activation of Fas ligand, and endoplasmic reticulum stress [6], [7], [8], [9]. Ultimately, apoptosis is characteristic of plaque progression and leads to instability, rupture, and subsequent clinical manifestation [10], [11], [12]. Thus, inhibition of the apoptotic process may lead to slowing or reversal of atheromatous plaque development [13].

Humanin (HN) is a 24-amino acid peptide encoded by mitochondrial 16S rRNA that was first discovered upon isolation from the occipital lobe of a patient with Alzheimer's disease [14], [15]. It was found to protect against neuronal apoptotic insult, specifically against amyloid-β toxicity and several familial disease-causing mutations [16], [17]. This protein has been shown to play a role in preventing cell death among various tissues outside of the nervous system as well [18], [19], [20], [21], [22]. We have recently demonstrated that HN is expressed in the endothelium of multiple vascular beds in humans and that its administration *ex vivo* results in decreased ROS production and apoptosis after oxidized LDL exposure in human aortic endothelial cells, a common inciting event in formation of the atherosclerotic plaque [20]. Moreover, we have demonstrated that HN exerts a protective effect on endothelial function and atherosclerotic progression in Apo E-deficient mice [23]. Thus, HN may play a protective role in atherosclerosis. The goal of this study was to test the hypotheses that HN is present in the atherosclerotic plaque in humans with expression correlating to apoptosis and symptomatic clinical presentation and whether it is localized to specific cell types.

## Methods

### Patients

This study was approved by the Mayo Foundation Institutional Review Board. Procedures were followed by institutional guidelines and written informed consent was obtained before surgery from all participants.

We studied 34 plaque specimens from patients undergoing carotid endarterectomy as previously described [8], [24], [25]. Surgical intervention was based on present clinical guidelines with the use of carotid artery imaging (ultrasound defining stenosis at 70–99%; magnetic resonance angiography defining 80–90% as severe and 90–99% as critical). Demographic data was obtained on all the patients by chart review with attention to cerebral ischemic events, coronary artery disease (CAD) risk factors, and medications. CAD was defined on the basis of a history of angina, myocardial infarction, coronary artery bypass graft, or percutaneous transluminal coronary angioplasty. Patients were deemed symptomatic if they showed a cerebral ischemic event 120 days prior to the surgery ipsilateral to the side of the collected plaque [26]. This included those with prior ischemic stroke (a neurologic episode lasting more than 24 hours) and transient ischemic attack (TIA, a reversible neurologic episode lasting less than 24 hours including amaurosis fugax). Patients having multiple events were categorized on the basis of their most severe event. Classification of asymptomatic patients was based on the absence of previous cerebral ischemic events.

### Carotid plaque specimens

After surgical excision, the plaques were cut in half at the site of maximal diameter. One half was fixed in formalin and embedded in paraffin for histology; the other half was immediately frozen and stored at −80°C for later tissue analysis [24], [25].

### Immunostaining for HN

Paraffin-embedded carotid plaques were immunostained according to protocol previously described, using double affinity purified rabbit anti-HN, developed at UCLA, in a concentration of 1 µg/mL [20], [25]. Normal rabbit immunoglobulin serum fractions were used as a negative control and testicular biopsy tissue served as a positive control [18], [20].

All stained specimens were sent to a Tissue and Cell Molecular Analysis core for scanning and conversion to digital microscopic images (Hamamatsu NanoZoomer Digital Pathology scanning microscope). These were computationally analyzed and quantified blindly as the percentage of stained area within the entire section using Olympus WebSlide Enterprise and Metamorph Meta Imaging Series 6.1 software.

### Western blotting for HN

Frozen tissue samples were prepared and analyzed by Western Blot as previously described [8], [25], [27]. Because HN is a very small peptide, we used a modified protocol in order to achieve band separation for radiographic densitometry. Carotid lysates were analyzed for protein content by Bradford Assay (Bio-Rad, CA). Equal amounts of protein were diluted in lysis buffer and reducing SDS loading buffer and resolved in a 16.5% Tris-Tricine SDS-polyacrylamide gel. Electrotransfer to nitrocellulose membrane was performed followed by blocking with 2% BSA/2% fat-free dry milk in TBS-T. Membranes were immunoblotted to detect HN (rabbit anti-HN, Abcam, 1∶500), then washed and incubated with secondary antibody conjugated to horseradish peroxidase (goat anti-rabbit IgG (H+L), Abcam, 1∶5000). A HN protein standard (CPC Scientific, CA) was run with the samples to correctly identify target bands and β-actin (rabbit anti-β-actin, Imgenex, 1∶5000) was used as an internal standard for protein loading. After development of the membranes with chemiluminescence (Thermo Scientific, IL) and exposure to X-ray film with a UVT 400-M transilluminator (IBI Kodak, NY), the resulting densitometric signals were analyzed using ImageJ software (National Institutes of Health) and presented as a ratio of HN/β-actin [25], [27].

### *In Situ* Detection of Apoptosis by Terminal Deoxynucleotidyl Transferase-Mediated dUTP Nick End-Labeling (TUNEL) Assay

Apoptosis was evaluated by the TUNEL method using a commercially available kit (Apoptag® Peroxidase In-Situ Apoptosis Detection Kit; Chemicon) [28] according to the vendor's instructions, as previously described [20], [25], using rat mammary gland tissue for positive control and omission of TdT enzyme as a negative control. Methyl green (Vector Laboratories, CA) was used for nuclear counterstain.

### Immunofluorescent staining for HN, CD68, α-actinin, vimentin, fascin, iNOS, and arginase 1

Immunofluorescence was used to detect the co-localization of HN (rabbit anti-HN, UCLA, 1∶250) with macrophages (mouse anti-CD68, Dako, 1∶100), smooth muscle cells (mouse anti-α-actinin, Abcam, 1∶100), fibroblasts (mouse anti-vimentin, Abcam, 1∶100), and dendritic cells (mouse anti-fascin, Dako, 1∶50), similar to protocol previously described [8]. Primary antibodies were pooled (HN+one cell marker) and incubated with the tissue overnight at 4°C followed by 30′ incubation with pooled secondary antibodies the next day at room temperature (Alexa Fluor 488 goat anti-rabbit IgG (H+L) and Alexa Fluor 568 goat anti-mouse IgG (H+L), Invitrogen, 1∶800). Slides were coverslipped with a DAPI-impregnated mounting medium (UltraCruz) and stored in the dark at 4°C. Normal rabbit and mouse immunoglobulin serum fractions were substituted for primary antibodies as negative controls.

We further characterized what state of macrophage polarization HN is associated with in regard to inducible nitric oxide synthase (iNOS)-positive proinflammatory (M1) macrophages or arginase 1-positive noninflammatory (M2) macrophages [29], [30]. In plaque sections stained with DAPI, single and double immunofluorescent stainings were performed with HN (rabbit anti-HN, UCLA, 1∶250) and either iNOS (Santa Cruz, 1∶100) or arginase 1 (Santa Cruz, 1∶100) conjugated antibodies. The number of double positive cells per field was quantified in 15–20 fields per slide which were then averaged in each sample over the entire patient group. Images were taken using a Zeiss LSM-510 confocal laser scanning microscope (Carl Zeiss MicroImaging, Inc).

### Immunofluorescent staining for MMP2 and MMP9

We also examined whether HN demonstrated co-localization with certain metalloproteinases recognized as inflammatory markers of plaque instability [8], [31]. The procedure was the same as described above with the exception of HN (rabbit anti-HN, UCLA, 1∶250) co-stained with either MMP2 (goat anti-MMP2, R&D Systems, 1∶100) or MMP9 (goat anti-MMP9, R&D Systems, 1∶100) primary antibodies followed by different secondary antibodies (Alexa Fluor 488 donkey anti-rabbit IgG (H+L) and Alexa Fluor 594 donkey anti-goat IgG (H+L), Invitrogen, 1∶800).

### Statistics

Statistical analysis was performed between groups using the *t* test for continuous variables and Fisher's exact test for categorical variables. Data is presented as either mean±SEM or percentage. Differences were considered significant when p<0.05.

## Results

Of the 34 patients included in this study, 22 were symptomatic and 12 were asymptomatic. Of the symptomatic patients, 12 had TIAs and 10 had strokes. There were no major differences in demographics between groups except that the symptomatic group (both TIA and stroke) had a higher average diastolic blood pressure and a lower prevalence of CAD compared to the asymptomatic group (see Table 1).

**Table 1:** Clinical Characteristics of Study Population.

	Asymptomatic (n = 12)	Symptomatic (n = 22)	TIA (n = 12)	Stroke (n = 10)
Age, y	73±2	71±2	69±3	72±3
Sex M/F, n	8/4	13/9	8/4	5/5
BMI, kg/m^2^	30.1±2.0	28.6±1.1	28.5±1.2	28.6±1.9
Systolic blood pressure, mm Hg	132.3±4.3	140.9±4.5	140.2±5.9	141.7±7.3
Diastolic blood pressure, mm Hg	68.4±2.2	77.2±1.7*	79.4±2.0*	74.7±2.7
Medical History				
CAD, n (%)	9 (75)	5 (22)*	3 (25)*	2 (20)*
Hypertension, n (%)	11 (92)	16 (73)	9 (75)	7 (70)
Diabetes, n (%)	2 (17)	3 (14)	2 (17)	1 (10)
Smoking History, n (%)	7 (58)	17 (77)	9 (75)	8 (80)
Hypercholesterolemia, n (%)	9 (75)	13 (59)	8 (67)	5 (50)
Lipid Profile				
Total Cholesterol, mg/dL	189.4±10.4	187.1±7.5	183.6±8.0	192.0±14.8
LDL, mg/dL	109.2±7.4	107.3±7.5	103.4±9.4	112.9±13.0
HDL, mg/dL	44.4±3.3	42.9±2.4	41.5±3.5	44.9±3.1
Medications				
Statins, n (%)	7 (58)	7 (32)	4 (33)	3 (30)
ACE-Inhibitors, n (%)	3 (25)	4 (18)	4 (33)	0 (0)
ARBs, n (%)	1( 8)	2 (9)	1 (8)	1 (10)
Aspirin, n (%)	10 (83)	19 (86)	12 (100)	7 (70)
Days since last event	0±0	79±21	71±27	85±35

BMI indicates body mass index; CAD indicates coronary artery disease; LDL indicates low density lipoprotein; HDL indicates high density lipoprotein; ARB indicates angiotensin II receptor blocker; Values are expressed as mean±SEM for continuous variables;

*p<0.05 vs. asymptomatic.

### HN is differentially expressed among clinically categorized plaques

**Figure 1:**
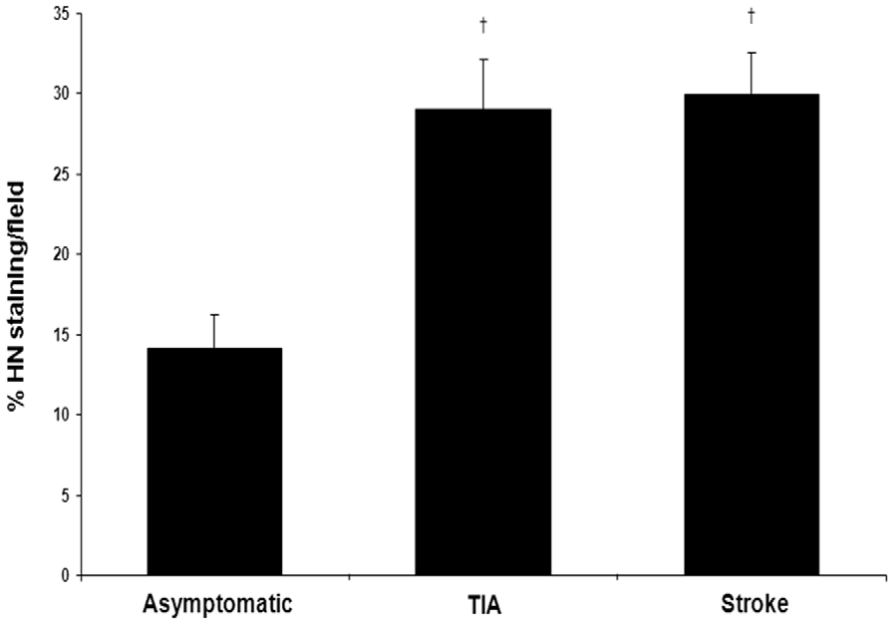
Histological immunoreactivity of HN is greater in symptomatic patients. HN expression is greater in carotid plaques from TIA (n = 12) and stroke (n = 10) symptomatic patients than in asymptomatic patients (n = 12; †p<0.001).

**Figure 2:**
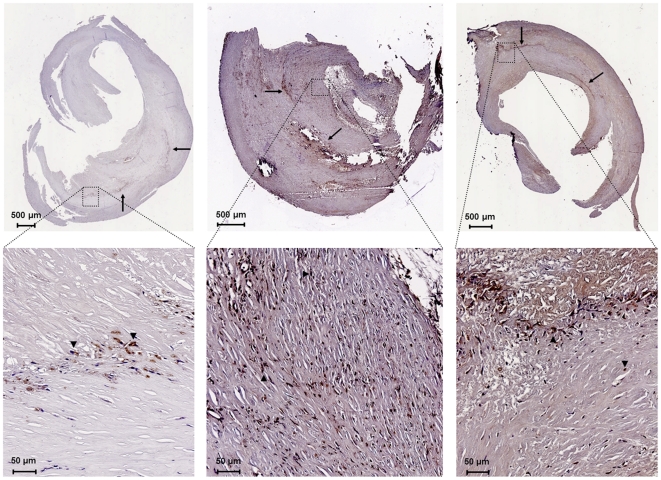
Representative immunostaining of HN. HN can be seen in carotid plaques (top row) from asymptomatic (left), TIA (middle), and stroke (right) patients. Increased magnification of these slides (bottom row) demonstrates intracellular localization (arrows indicate examples of staining).

**Figure 3:**
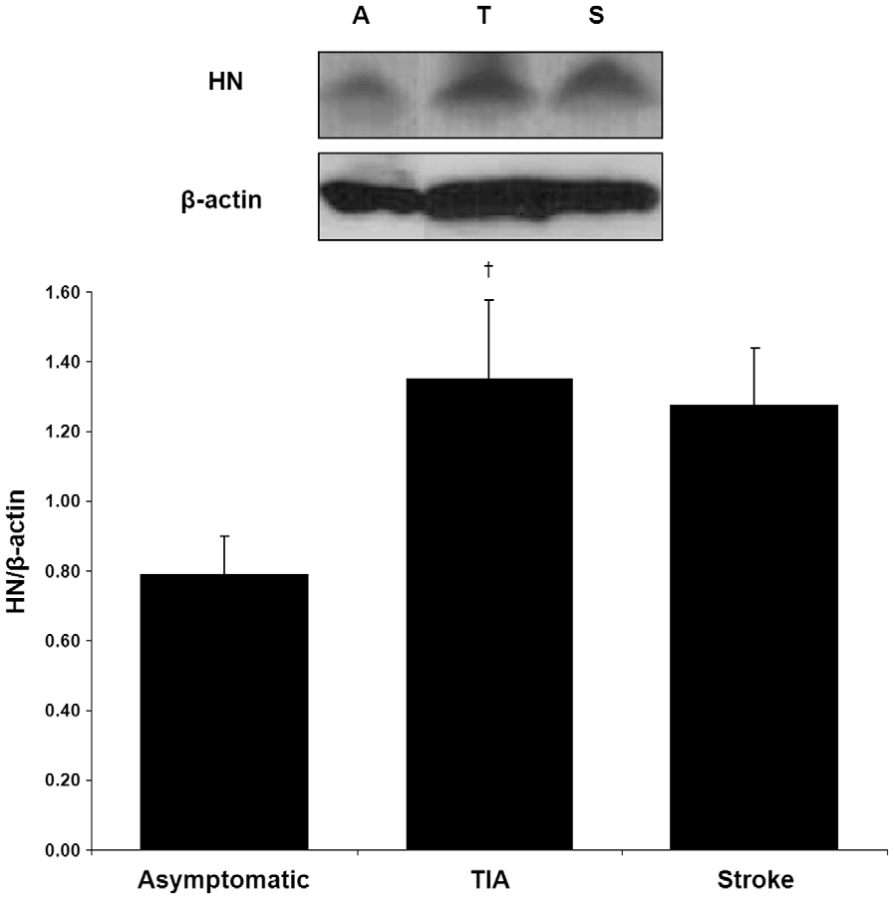
HN protein blotting is greater in symptomatic patients. Western Blot demonstrates amount of HN is greater in carotid plaques of symptomatic patients than in asymptomatic patients (combined symptomatic not depicted, p<0.01). Representative immunoblot shown above. Results expressed as the ratio of HN and β-actin densitometric signals (†p<0.05 vs. asymptomatic).

**Figure 4:**
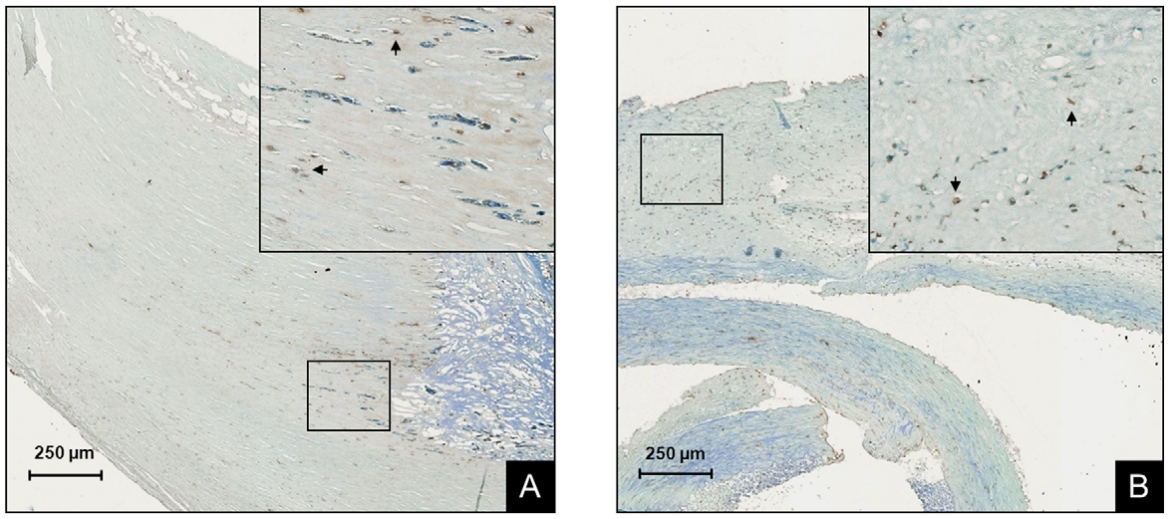
Cellular apoptosis is greater in plaques of symptomatic patients. Representative TUNEL staining can been seen in carotid plaques from (A) asymptomatic and (B) symptomatic patients. Inserts show magnification of the black boxes in the main figure and arrows indicate brown apoptotic nuclei (original magnification 200×). Proportion of apoptotic nuclei was greater in plaques of symptomatic patients compared to asymptomatic (p<0.01).

The presence of HN was greater in the unstable plaques of symptomatic patients (Figure 1). Immunostaining and quantification of carotid plaques demonstrated higher levels in the symptomatic group compared to the asymptomatic group (29.42±2.05 vs. 14.14±2.13% of plaque area, p<0.0001). The increased amount of HN in the symptomatic group was attributable equally to the TIA and stroke subgroups (Figure 1; 29.00±3.18 and 29.92±2.60%, respectively, p<0.001). HN seemed to be diffusely localized throughout the plaque, most notably in regions of increased cellularity (Figure 2). This data obtained from histology was further confirmed by Western blot analysis. The expression of HN in the plaque was greater in the symptomatic group compared to the asymptomatic group (density ratio of HN/β-actin 1.32±0.14 vs. 0.79±0.11, p<0.01). In this case, the TIA subgroup was significantly greater than the asymptomatic group (Figure 3; density ratio of HN/β-actin 1.35±0.23 vs. 0.79±0.11, p<0.05) while the stroke subgroup showed a trend (density ratio of HN/β-actin 1.28±0.17 vs. 0.79±0.11, p = 0.08). Interestingly, linear regression of the symptomatic plaque data revealed increased expression of HN correlating with the number of days since the ischemic event (∼5% increase in HN staining per 100 days after the event, R = 0.50, p = 0.03, data not shown). Similarly, we found that TUNEL staining revealed a higher proportion of apoptotic nuclei in these same plaques of symptomatic patients compared to asymptomatic (Figure 4; 68.25±3.61 vs. 33.46±4.46% of nuclei, p<0.01).

### HN is localized to cells of the atherosclerotic plaque notably involved with an inflammatory state

**Figure 5:**
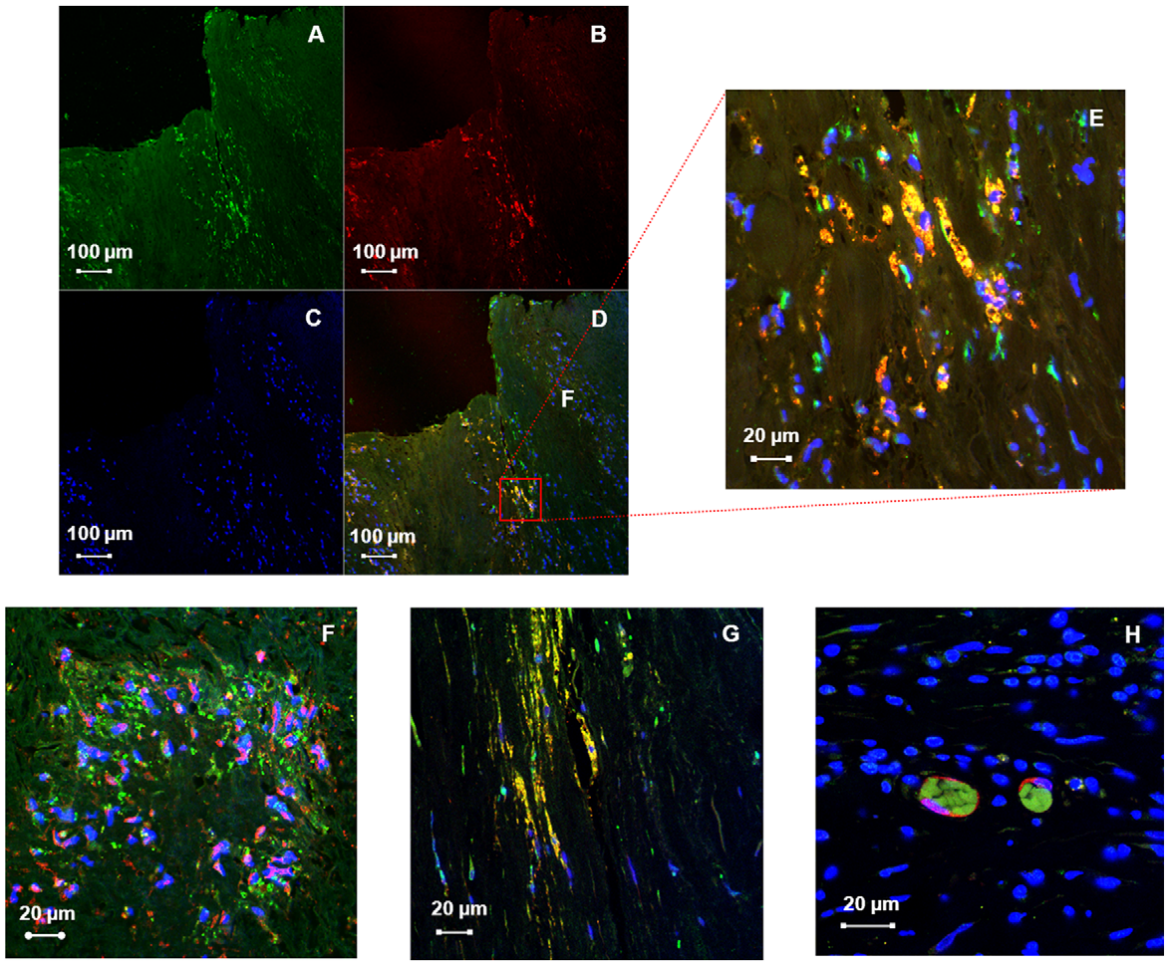
HN is localized to multiple cells of the atheromatous plaque. Immunofluorescence of the carotid plaque to detect the presence of (A) HN with (B) CD68. (C) Nuclei can be visualized using a DAPI counterstain. (D) Merging of these images demonstrates co-localization of HN with plaque macrophages, (E) seen better with increased magnification. Merged images below demonstrate co-localization of HN with (F) smooth muscle α-actinin, (G) fibroblast vimentin, and (H) dendritic cell fascin.

Immunostaining revealed intracellular localization of HN with light microscopy. To ascertain which cells are contributing to the expression of HN in the plaque, we used double immunofluorescence labeling and confocal microscopy to visualize the co-localization of HN with known cell markers. HN was co-expressed with CD68(+) macrophages, α-actinin(+) smooth muscle cells, vimentin(+) fibroblasts, and fascin(+) dendritic cells (Figure 5).

**Figure 6:**
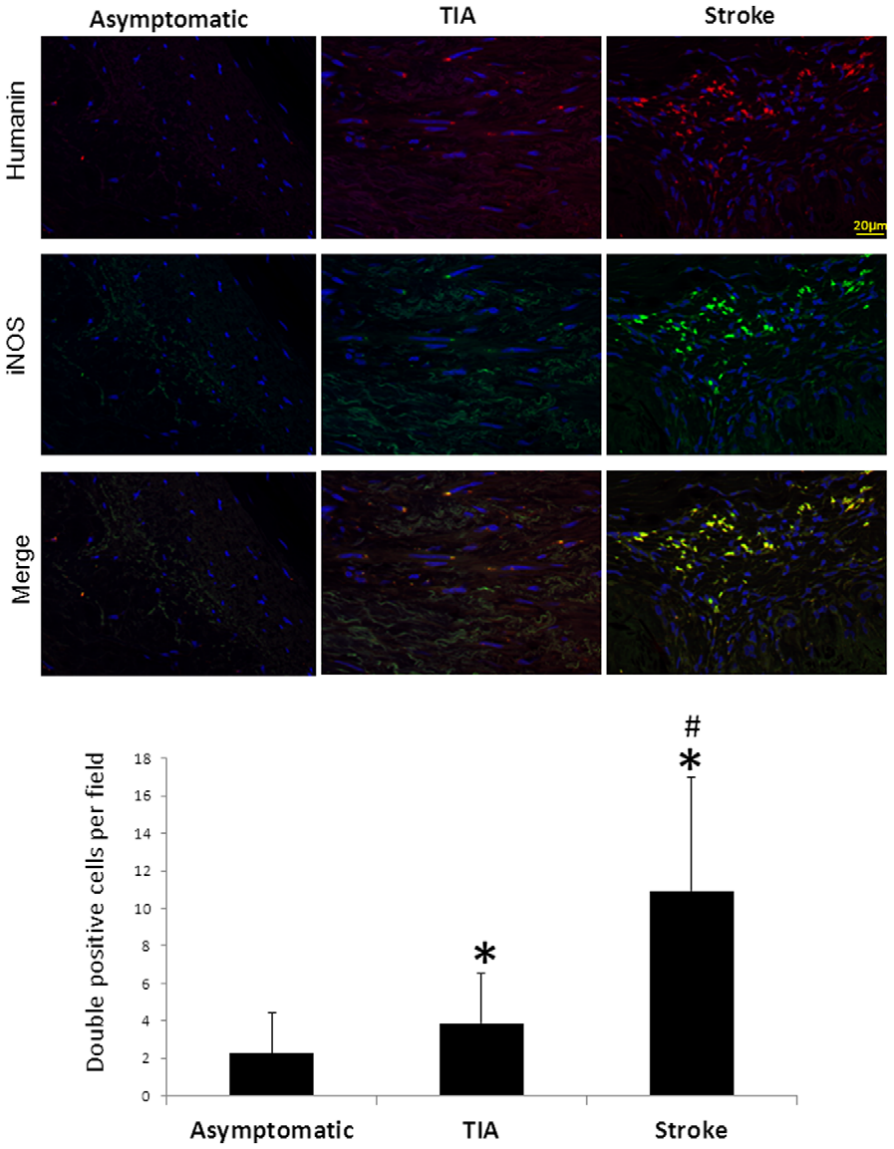
HN is expressed in M1 phase macrophages. Immunofluorescence reveals the presence of more HN-secreting macrophages in the M1 polarization phase (indicated by iNOS) in symptomatic patients (*p<0.05 vs. asymptomatic, #p<0.05 vs. TIA).

**Figure 7:**
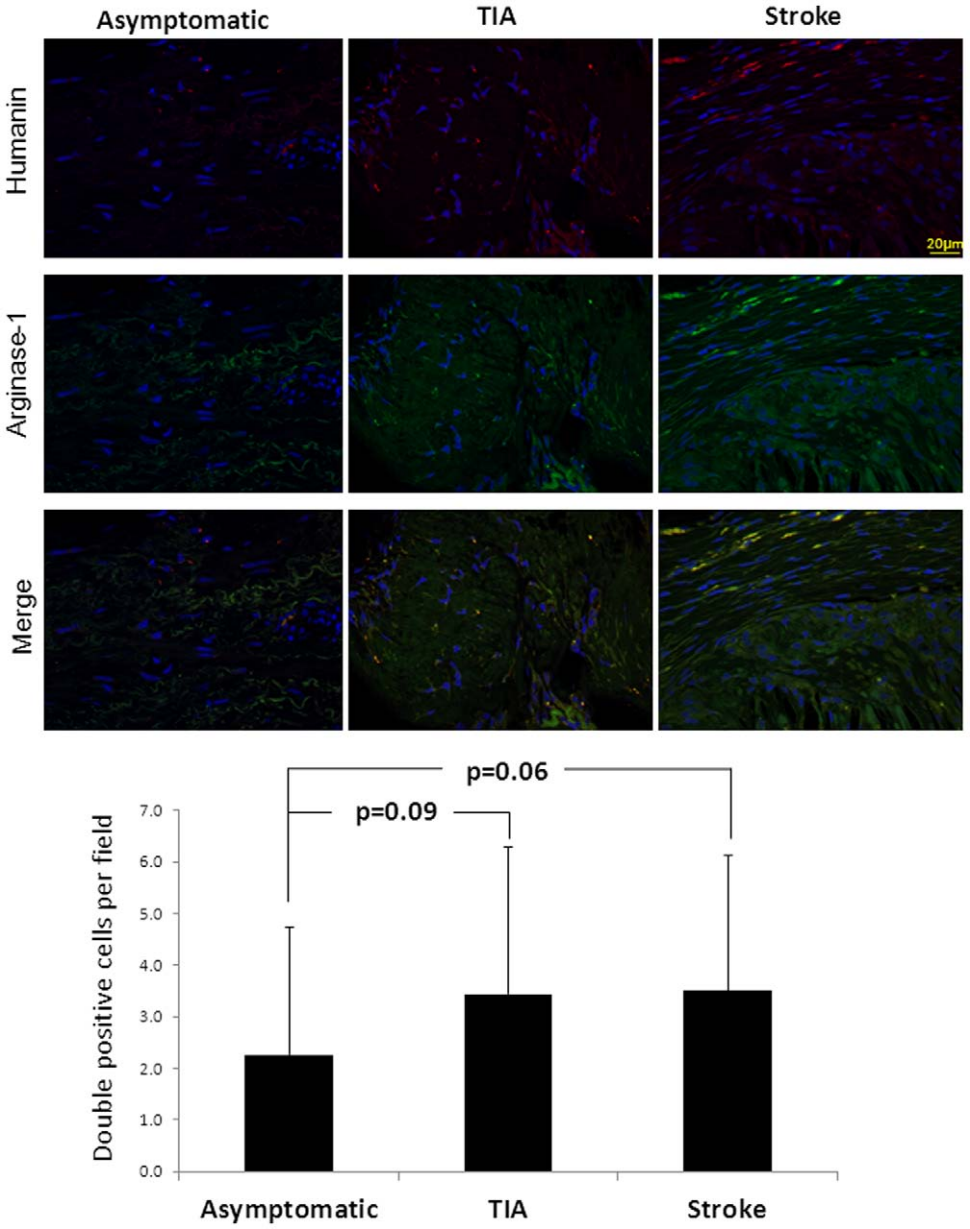
HN is expressed in M2 phase macrophages. HN-secreting macrophages in the M2 polarization phase (indicated by arginase-1) were present, but less in number than HN-secreting M1 macrophages. No differences were seen between groups.

**Figure 8:**
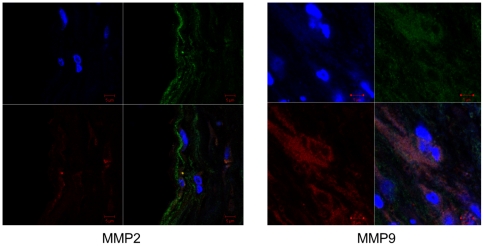
HN co-localizes with MMP2 and MMP9 in the plaque. (Left) HN (green) co-localizing with MMP2 (red) in the intima with DAPI counterstain (blue). (Right) Similar staining can be seen with MMP9 (red) in the media.

In addition, HN was found to be expressed in both M1 and M2 phase macrophages. Interestingly, the presence of proinflammatory M1 macrophages co-stained with HN was greater in symptomatic patients compared with asymptomatic patients (Figures 6 and 7; p<0.05). However, no significant difference was noted among patient groups for HN-positive M2 macrophages. Along with these findings, MMP2 was co-localized with HN in the intima and media, while MMP9 was predominant mostly in the media (Figure 8).

## Discussion

The current study demonstrates for the first time that the HN protein is present in carotid atherosclerotic plaques and that its expression is greater in patients with symptomatic disease compared to those with asymptomatic disease. The immunoreactivity of HN for both blotting and staining was nearly two-fold in the symptomatic group than that of the asymptomatic group. Additionally, we found the amount of apoptosis in these symptomatic plaques is greater, consistent with prior studies demonstrating that factors involved with the progression and instability of the atheromatous plaque (such as inflammation, reduction of proteosome activity, and oxidative stress) correlate with a symptomatic history involving an ischemic event [8], [12], [25]. A large body of evidence supports the propensity of unstable plaques to result in significant, possibly life-threatening, clinical manifestations and the characteristics of these vulnerable plaques have been described extensively [32], [33], [34]. Consequently, studies investigating cytoprotection and mitigating the potentially adverse effects of apoptosis (in regard to both endothelial dysfunction and the late-stage necrotic atheroma) as a means to prevent atherosclerotic complications may be of considerable value [13], [35], [36], [37]. The beneficial effect of inhibiting apoptosis in atherosclerosis is controversial, however, as it may alternatively help reduce cellularity of early plaque lesions in conjunction with efferocytosis [38]. Given these observations we present here an endogenously expressed peptide in the atherosclerotic plaque that is widely shown elsewhere to have cytoprotective properties with a notable role of preventing apoptosis.

Evidence of these properties have been seen in CNS neurons [39], [40], cerebrovascular smooth muscle cells [41], testicular germ cells [18], [42], pancreatic beta cells [21], and skeletal muscle cells [19]. HN is found to modulate cellular apoptosis by binding several Bcl-2/Bax family proteins and inhibiting their translocation to the mitochondrion, preventing the subsequent release of cytochrome *c* into the cytosol [43], [44], [45]. HN also binds and antagonizes the pro-apoptotic molecule IGFBP-3 and binds an extracellular membrane receptor (composed of the CNTF-R, WSX-a, and GP130 subunits), which subsequently activates the JAK2/STAT3 pathway [46], [47], [48], [49], [50], [51]. Because studies have demonstrated that it can protect against memory impairment and ischemic injury [52], [53], HN and synthetic HN-analogues are currently under investigation as Alzheimer's disease treatments [49], [54], [55]. Interestingly, HN has also been shown to be a metabolo-protective factor and is able to normalize blood sugar in diabetic ZDF rats through a hypothalamic mechanism involving STAT3 activation [56].

The role of HN in the cardiovascular system is also now emerging. HN decreases the myocardial infarct size in an experimental model of ischemia and reperfusion in mice [22] and prevents ROS production and death of human aortic endothelial cells exposed to Ox-LDL [20]. The latter is of note because Ox-LDL itself results from the penetration of LDL across the damaged endothelial cell layer into the subendothelial space where it is converted by ROS produced from endothelial and smooth muscle cells. Ox-LDL then propagates the formation of ROS leading to oxidative stress, inflammation, and the formation of atherosclerotic plaque [57], [58], [59]. Thus, the histological finding of HN in the plaque extends our previous observations and supports a potential role for HN in atherosclerosis, possibly as an endogenous response to the inflammatory and apoptotic processes within the atheromatous plaque.

At present, the mechanism leading to higher expression of HN in plaques of symptomatic patients remains speculative, particularly because progression of the atheromatous plaque is multifactorial. One possibility is that HN is being produced as part of an injury response mechanism. Since apoptosis is a natural process in late-stage atherosclerosis contributing to formation of a necrotic core and unstable plaque, the expression of HN might be a defense mechanism to slow progression of the disease. But since unstable plaques demonstrate higher levels of HN, this compensatory response may not be sufficient to withstand sustained insult and consequently, eventual ischemic events result. Another possibility may be that HN is involved with the stabilization of carotid plaques following stroke, a process notably associated with the decrease of caspase-3, a marker of apoptosis [60], [61]. Our finding that HN in the plaque modestly increases with time after an ischemic event may be associated with this. Further investigation correlating markers of apoptotic pathways in the carotid plaque with the expression of HN, particularly with those that bind HN directly including the pro-apoptotic molecule IGFBP-3 and extracellular JAK2/STAT3 pathway membrane receptor (composed of the CNTF-R, WSX-a, and GP130 subunits) [46], [47], [50], may help answer these questions.

We also demonstrated intracellular expression of HN in macrophages, smooth muscle cells, fibroblasts, and dendritic cells (it should be noted that distinguishing smooth muscle cells from fibroblasts may be difficult in certain instances because myofibroblasts are reported in the atherosclerotic plaque) [62], [63]. A couple of observations in the literature are consistent with these findings: 1) exogenous HN has been shown to prevent apoptosis in smooth muscle cells of CNS vasculature [41] and 2) HN acts as an agonist on the formyl peptide receptor-like 2 (FPRL2) found on the extracellular membrane surface of macrophages and dendritic cells [64], [65]. Moreover, HN originates from the mitochondrial genome, which may explain its ubiquitous expression [39]. In addition, HN was found to be expressed by both states of macrophage polarization, though predominantly more expressed by proinflammatory M1 macrophages in symptomatic patients. HN also co-localized with MMP2 and MMP9, inflammatory markers with the ability for enzymatic remodeling of the fibrous cap as well as signaling vascular smooth myocytic migration [31]. Taken together, these data suggest that these infiltrating cells of the atheroma may be producing and secreting HN to exert protective effects via an intracellular, autocrine, and/or paracrine mechanism, possibly involving the mitochondrial oxidative/apoptotic response to oxidant-induced mitochondrial dysfunction in atherogenesis [20], [39], [66], [67]. In line with this, recent reports have discussed the possibility of mitochondria-specific antioxidative mechanisms to treat atherosclerosis by decreasing mitochondrial damage and permeability [68], [69]. This ultimately would inhibit cytochrome *c* release and oxidant-induced apoptosis, interestingly the same net benefit imparted by the mitochondrial-derived peptide, HN [43], [44], [45], [48], [49], [70].

In summary, the current study demonstrates that HN is present in carotid atherosclerotic plaques and that its higher expression is associated with cellular apoptosis and a clinical symptomatic history of ischemia. Moreover, we were able to localize its expression within various cells of the plaque, notably those associated with states of inflammation. These data support a role for HN in atherosclerosis.
