# Correction: Humanin, a Cytoprotective Peptide, Is Expressed in Carotid Artherosclerotic Plaques in Humans

**DOI:** 10.1371/annotation/1a60b239-181f-4d5f-9a37-3d3b04949954

**Published:** 2012-03-22

**Authors:** David G. Zacharias, Sung Gyun Kim, Alfonso Eirin Massat, Adi R. Bachar, Yun K. Oh, Joerg Herrmann, Martin Rodriguez-Porcel, Pinchas Cohen, Lilach O. Lerman, Amir Lerman

There was a typographical error in the title. The correct title is "Humanin, a Cytoprotective Peptide, Is Expressed in Carotid Atherosclerotic Plaques in Humans". The correct citation is: Zacharias DG, Kim SG, Massat AE, Bachar AR, Oh YK, et al. (2012) Humanin, a Cytoprotective Peptide, Is Expressed in Carotid Atherosclerotic Plaques in Humans. PLoS ONE 7(2): e31065. doi:10.1371/journal.pone.0031065 

